# Periodontal Health Knowledge and Oral Health-Related Quality of Life in Caribbean Adults

**DOI:** 10.3290/j.ohpd.b4836035

**Published:** 2024-01-15

**Authors:** James R. Collins, Sona Rivas-Tumanyan, Arvind Babu Rajendra Santosh, Augusto Elias Boneta

**Affiliations:** a Professor, Department of Periodontology, School of Dentistry, Pontificia Universidad Católica Madre y Maestra (PUCMM), Santo Domingo, Dominican Republic. Principal investigator Dominican Republic, developed concepts, design, and intellectual content of the study and manuscript, involved in the preparation, editing, and review of the manuscript and is the corresponding author.; b Associate Professor, School of Dental Medicine, Medical Sciences Campus, University of Puerto Rico (UPR SDM), San Juan, Puerto Rico. Data/statistical analysis, manuscript review.; c Professor, School of Dentistry, Faculty of Medical Sciences, The University of the West Indies, Mona Campus, Jamaica, West Indies; Department of Oral Medicine, Faculty of Dental Medicine, Universitas Airlangga, Indonesia. Principal investigator Jamaica, Concepts, design, intellectual content of the study and manuscript, reviewed the manuscript.; d Professor, School of Dental Medicine, Medical Sciences Campus, University of Puerto Rico (UPR SDM), San Juan, Puerto Rico. Principal investigator Puerto Rico, concepts, design, intellectual content of the study and manuscript, reviewed the manuscript.

**Keywords:** Caribbean adults, OHIP-14 questionnaire, oral health, patient education, quality of life

## Abstract

**Purpose:**

To identify the relationship between periodontal health knowledge and oral health-related quality of life among Caribbean adults.

**Materials and Methods:**

A cross-sectional study was conducted in a representative sample from 3 Caribbean cities (weighted N=1805). Participants completed a questionnaire on oral health knowledge, hygiene habits, and other practices, as well as the Oral Health Impact Profile-14 (OHIP-14) questionnaire. The associations between knowledge and habits and OHIP-14 score and its tertiles were evaluated using negative binomial and multinomial logistic regression models, respectively, adjusting for confounders. Odds ratios and regression coefficients were reported.

**Results:**

Participants reporting none, little, and adequate knowledge about gum health had higher odds of being in the worst tertile for OHRQoL, compared to those reporting “good knowledge” (ORnone vs good=2.38, 95% CI: 1.59–3.54; ORlittle vs good=1.82, 95% CI: 1.19–2.78; ORadequate vs good=1.68, 95% CI: 1.11–2.57). Participants reporting toothbrushing≥twice/day were less likely to be in the worst tertile for OHRQoL, compared to those brushing less often (OR=0.67, 95% CI: 0.48–0.92). Self-reported gum bleeding was associated with double the odds of being in the worse tertile (OR=2.03, 95% CI: 1.60–2.58).

**Conclusion:**

According to the findings of this study, periodontal health knowledge is associated with reduced OHRQoL in Caribbean Adults. In addition, the frequency of brushing and the self-reported gum bleeding was related to a worse quality of life (QoL) level.

The World Dental Federation (FDI) has defined oral health as “multifaceted and includes the ability to speak, smile, smell, taste, touch, chew, swallow, and convey a range of emotions through facial expressions with confidence and without pain, discomfort, and disease of the craniofacial complex”.^9^ This definition encompasses the physical, psychological, social and mental well-being of the individual, which are considered essential to improve the Oral Health-Related Quality of Life (OHRQoL) of the people. However, to obtain this health status and well-being, it is necessary that the individual have knowledge and attitudes that allow her/him to perform adequate oral health practices. Recent research has demonstrated that oral health knowledge, practice, and self-rated oral health directly and positively affect OHRQoL. Kwon et al^12^ evaluated the relationship between oral health knowledge (OHK) and oral health-related quality of life among older adults. The researchers concluded that poor oral health knowledge was statistically significantly associated with participants over the age of 75years (OR=1.9; 95% CI: 1.15–3.16), highschool education or less (OR=10.8; 95% CI: 5.92–19.84), minority ethnicity (OR=7.3; 95% CI: 4.27–12.61), and reading ability less than “excellent” (OR=7.27; 95% CI: 4.35–12.14). In addition, they were able to show that participants with poor OHK were 5.17 times more likely to be identified with higher OHIP severity scores.

Recently, considerable global attention has been paid to the importance of oral health for general health.^7^ Epidemiological, clinical and experimental studies have demonstrated the association between oral and systemic diseases (metabolic syndrome, pregnancy complications, rheumatoid arthritis, cognitive disorders including Alzheimer’s disease, cardiovascular diseases and even some types of cancers).5,17,18 Akl et al^1^ conducted a systematic review to investigate the knowledge and awareness of patients with major systemic conditions about associations between oral conditions and their illness. Twenty-four studies from 14 different countries were included in this systematic review. The study stated that patients with major systemic conditions have poor knowledge and awareness of the relationship between oral health and their condition.^1^ Interestingly, dentists and the media were the most common sources of information. These results show the importance of integrating the patient’s education about the oral-systemic disease relationship into the clinical practice, in order to help reduce the prevalence of systemic diseases on a global scale.^1^

Knowledge of oral health is considered essential to develop healthy practices and attitudes; this has been demonstrated through studies which show a correlation between greater knowledge and better oral health.^19^^,^^26^ A previous study defined oral health literacy as “the degree to which individuals have the capacity to obtain, process and understand basic health information and services needed to make appropriate oral health decisions”.^16^

Periodontal status, levels of education and OHRQoL were associated in a population of Caribbean adults.^6^ This recent epidemiological study showed that lower levels of education and visiting the dentist only when there was a problem were associated with higher odds of presenting a worse OHRQoL.^6^ Yactayo-Alburquerque et al^25^ performed a systematic review of studies conducted in Latin America and the Caribbean (LAC) to assess the impact of oral diseases on oral health-related quality of life (OHRQoL). The study designs included 32 cross-sectional, 2 cohort and 6 case-control studies. The authors concluded that almost all studies reported an impact on OHRQoL in children, adolescents and adults with oral diseases.^25^

Epidemiological studies aimed at investigating the oral health knowledge, attitudes and practices of certain populations are considered essential to understand the distribution of oral diseases in vulnerable populations, which allows the creation of effective strategies to develop innovative models for the promotion of good oral health.^21^^,^^22^ They also allow and encourage the development of new methods to stimulate healthy attitudes in individuals and their families. In a multicentric study (Jamaica, Dominican Republic and Puerto Rico), Elias-Boneta et al^8^ stated the prevalence of moderate gingival inflammation to be 81.9%. However, there are no studies in this region on association of oral health knowledge and practices with OHRQoL. Thus there is considerable need to understand the knowledge, attitude and practices of oral hygiene among Caribbean adults and relate them with factors that either affect or alter oral health status and the quality of their life.

In the present survey, our hypothesis was that adults who present poor periodontal health knowledge, attitudes and practices are more likely to have worse oral health-related quality of life. Thus, the aim of the current study was to identify through a survey the periodontal health knowledge, attitudes and practices among Caribbean adults. The study also aimed to demonstrate oral hygiene habits and factors affecting oral health-related quality of life (OHRQoL) in the same population.

## MATERIALS AND METHODS

### Ethical Approval 

Ethical approval was obtained from the Ethics and Medico-Legal Affairs Panel of the Ministry of Health and the UWI Ethics Committee of the University of the West Indies, Mona Campus, Jamaica (Protocol #248, 15/16); the Bioethics Committee of the Faculty of Health Sciences of the Pontificia Universidad Católica Madre y Maestra and the National Council of Bioethics in Health of the Ministry of Public Health in the Dominican Republic (Protocol #042–2016); and in Puerto Rico, the Institutional Review Board of the University of Puerto Rico, Medical Sciences Campus (UPR-MSC) (Protocol #360216).

### Subjects and Design

The description of the population under study has been published previously.^8^ Briefly, a cross-sectional study was conducted among 1848 adults (weighted N=1830) from 3 Caribbean cities: Kingston, Jamaica; Santo Domingo, Dominican Republic; and San Juan, Puerto Rico. Participants were recruited in 8 geographical clusters (with 76-77 participants each) in each city, using a systematic random sampling technique. Members of the general population who expressed an interest in participating and met the inclusion criteria (good general health [ASA I & II], 18years of age or older, presence of at least 4 permanent natural teeth), consented to participate in the study. Participants were excluded if pregnant or breastfeeding, had undergone extensive prosthodontic treatment (partial removable dentures and/or fixed prosthodontics), were wearing orthodontic appliances (except retainers) and/or presented gingival purulent exudate, tooth mobility, and/or extensive loss of periodontal attachment or alveolar bone. Participants needing prophylactic antibiotic therapy, who were on anticoagulant medication/treatment (except aspirin, but including nifedipine, cyclosporine, or phenytoin), or taking any other prescription medicines that might interfere with the study outcome were also excluded. Non-eligible candidates received a general oral screening and were offered oral health advice and referrals, where necessary.

Before starting the study, the researchers met at the Medical Sciences Campus of the University of Puerto Rico, where they discussed the protocol and the questionnaire to be implemented. After discussing and agreeing on all the details of the survey, the researchers were calibrated to achieve acceptable agreement in their measurements of gingival inflammation. The clinical examiners of this study were calibrated after didactic training on the gingival indices and diagnostic criteria for gingival and periodontal diseases. The participants included both periodontally healthy individuals and patients with a full range of periodontal conditions. The inter-examiner variations were statistically calculated through Spearman’s correlation coefficient test. The mean gingival index (GI) taken by the clinical examiners were averaged and ranged from 0.43 to 0.71 (p<0.05).

### Survey Instruments and Data Collection

Informed consent was obtained from each participant before the administration of the questionnaire by trained study personnel. The questionnaire items were selected from two validated questionnaires in English and Spanish: Medical History/ Oral Health (M/OH) and Oral Health Impact Profile (OHIP) questionnaires.^12^^,^^13^ The M/OH questionnaire collected information on socio-demographics, general health, dental visits, oral hygiene habits and knowledge, the frequency of dental visits, prosthesis use/hygiene, and smoking. The short form of the Oral Health Impact Profile (OHIP-14) index was used to evaluate OHRQoL and collected information about oral hygiene habits, smoking, educational level, and self-reported gingival bleeding.^19^ Both questionnaires were administered by study staff in an interview setting. The English versions of the study questionnaires are included as supplemental material to this manuscript (Supplemental I). Participants who completed the interview received advice about appropriate oral health care and were referred for oral/dental treatment, as required.

### Statistical Analysis

After the exclusion of 9 participants who did not complete the OHIP questionnaire, the weighted sample size was 1821. We further excluded participants with missing values for self-assessed knowledge (N=5), mouthwash use (N=1), toothbrushing frequency (N=6), self-reported gum bleeding (N=1), smoking (N=3), and level of education (N=4), resulting in the final weighted sample size of 1805. Participants with missing information on the frequency of dental visits were grouped under a separate category (N=81). The analysis of practices when experiencing gum bleeding was limited to participants who reported gum bleeding in the previous question, resulting in a weighted sample size of 1040 for this sub-analysis.

All analyses accounted for clustering in design and were weighted using normalised weights. The continuous summary OHIP score, as well as its categorical version (tertiles of OHIP), were used as study outcomes, with the upper third tertile corresponding to the worst levels of QoL. In the descriptive analysis, the distribution of the summary OHIP score and its tertiles was compared across the categories of potential predictors. The associations between predictors (such as oral health knowledge and hygiene practices) and the study outcomes were further evaluated using negative binomial regression (for the continuous OHIP score) and multinomial logistic regression (for tertiles of OHIP score) models. Models were adjusted for potential confounders: participants’ age (years), biological sex (male, female), smoking (3 categories: never, past, current), education (university, technical, none/basic), frequency of dental visits (only when there is a problem, at least once a year, never, missing), number of missing teeth, and location (Kingston, San Juan, Santo Domingo). Correlation matrices of independent variables were examined for potential collinearity between the predictors; no potential collinearity issues were identified. Regression coefficients (standard errors), and their exponentiated values (95% confidence intervals) were reported for the OHIP score; odds ratios (95% confidence intervals) were reported for tertiles of the OHIP score.

All analyses were conducted at the 0.05 level of statistical significance, using SAS statistical software version 9.4 (SAS Institute; Cary, NC, USA).

## RESULTS

A total of 1823 adults (weighted N=1805) from Santo Domingo (WtN=607), San Juan (WtN=604), and Kingston (WtN=594) were included in this analysis. Participant age ranged between 18 and 96, with a weighted mean of 40.32 (SE: 0.15) years; 45.76% were males (Table 1). Most of the participants were never smokers (Table 1: 64.74%), had middle/technical education (54.56%) and reported visiting the dentist only when they had a problem (59.17%). Among all participants, the average oral health-related quality of life score was 7.2 (SE: 0.17), with a median of 4 points (Table 2). About 45% of participants who self-assessed their knowledge of gum health as being “good” were in the lowest tertile for OHIP, indicating a better quality of life (QoL) (Table 2). On the other hand, 41% of those who indicated no knowledge of gum health were in the top tertile for OHIP (poorest quality of life). Participants who reported flossing were more likely to be in the lower tertile of OHIP (38.35%), and a similar trend was seen among those who reported toothbrushing twice/day or more often (35.91% in the lowest tertile). 40.66% of those who reported toothpick use were in the top (worst) tertile for OHIP. Participants who reported gum bleeding were more likely to be in the top tertile (39.19%), compared to those who reported no bleeding (29.34%).

**Table 1:** Distribution of oral health-related quality of life (OHIP) scores, sociodemographic and health-related variables, among all participants, and by tertiles of OHIP scores

	All participants (WtN=1805)	Participants in OHIP Tertile 1 (WtN=642)	Participants in OHIP Tertile 2 (WtN=531)	Participants in OHIP Tertile 3 (WtN=632)
**OHIP scores**				
Mean (SD)	7.20 (7.97)	0.80 (0.92)	4.89 (1.28)	15.63 (7.95)
Median (IQR)	4.05 (1.14–9.56)	0 (0–1.28)	4.13 (3.32–5.53)	12.57 (9.23–18.46)
Range (min-max)	(0–53)	(0–2)	(3–7)	(8–53)
**Age, years**				
Mean (SD)	40.32 (15.28)	40.14 (15.58)	39.77 (15.13)	40.97 (15.09)
Median (IQR)	37.91 (27.00–50.90)	37.22 (26.84–50.82)	36.87 (26.57–49.82)	38.97 (27.72–51.80)
Male, n (%)	826 (45.76%)	326 (50.79%)	248 (46.77%)	251 (39.80%)
**Smoking, n (%)**				
Never	1168 (64.74%)	439 (68.39%)	350 (65.96%)	379 (60.00%)
Past	279 (15.45%)	105 (16.32%)	82 (15.45%)	92 (14.56%)
Current	358 (19.82%)	98 (15.29%)	99 (18.59%)	161 (25.44%)
**Education, n (%)**				
None/basic	265 (14.69%)	79 (12. 30%)	70 (13.14%)	116 (18.41%)
Middle/technical	985 (54.56%)	341 (53.12%)	294 (55.40%)	350 (55.31%)
University	555 (30.76%)	222 (34.58%)	167 (31.46%)	166 (26.28%)
**Any disease/condition, n (%)**	678 (37.57%)	228 (35.52%)	182 (34.29%)	268 (42.42%)
**Frequency of dental visits, n (%)**				
Only when there is a problem	1068 (59.17%)	343 (53.51%)	310 (58.40%)	414 (65.57%)
Never	60 (3.34%)	27 (4.28%)	12 (2.28%)	21 (3.26%)
At least once a year	597 (33.09%)	239 (37.17%)	191 (35.92%)	168 (26.57%)
Missing	81 (4.47%)	32 (5.04%)	18 (3.40%)	29 (4.60%)
**Number of missing teeth**				
Mean (SD)	3.69 (4.90)	2.97 (4.45)	3.60 (4.78)	4.49 (5.31)
Median (IQR)	1.30 (0.00–4.82)	0.66 (0.00–3.51)	1.39 (0.00–4.62)	1.94 (0.00–6.47)
**City, country of residence, n (%)**				
Kingston, Jamaica	596 (32.89%)	197 (30.69%)	174 (32.75%)	223 (35.23%)
San Juan, Puerto Rico	604 (33.49%)	231 (36.01%)	189 (35.63%)	184 (29.12%)
Santo Domingo, Dominican Republic	607 (33.63%)	214 (33.30%)	168 (31.62%)	225 (35.64%)

**Table 2:** Distribution of oral health-related quality of life scores and tertile categories, among all participants and within categories defined by responses to knowledge and behaviour questions

	WtN	Mean (SD)	Median (Q1–Q3)	Tertile 1 WtN (%)	Tertile 2 WtN (%)	Tertile 3 WtN (%)
Among all participants	1805	7.20 (7.97)	4.05 (1.14–9.56)	642 (35.56%)	531 (29.42%)	632 (35.02%)
1. Self–assessed knowledge about gum health						
None	485	8.24 (8.42)	5.34 (1.33–11.88)	159 (32.80%)	125 (25.82%)	201 (41.38%)
Little	666	7.39 (8.05)	4.53 (1.22–9.85)	229 (34.41%)	195 (29.22%)	242 (36.38%)
Adequate	453	6.34 (7.11)	3.68 (1.09–8.06)	163 (35. 98%)	152 (33.67%)	137 (30.35%)
Good	201	6.00 (8.13)	3.13 (0.10–7.27)	91 (45.09%)	59 (29.22%)	52 (25.69%)
2. In your opinion, gums bleed because… (mark all that apply)						
Don’t know: marked	701	6.89 (8.09)	3.75 (0.28–9.39)	271 (38.64%)	191 (27.18%)	240 (34.17%)
not marked	1104	7.40 (7.89)	4.51 (1.33–9.67)	371 (33.60%)	340 (30.84%)	392 (35.56%)
Bad toothbrushing/hygiene: marked	463	7.33 (7.38)	4.59 (1.52–9.46)	145 (31.41%)	154 (33.33%)	163 (35.26%)
not marked	1342	7.16 (8.17)	3.95 (1.00–9.60)	496 (36.99%)	377 (28.07%)	469 (34.94%)
Smoking: marked	34	8.72 (11.54)	4.49 (0.19–8.95)	13 (38.30%)	11 (32.32%)	10 (29.38%)
not marked	1771	7.17 (7.89)	4.04 (1.16–9.55)	629 (35.51%)	520 (29.37%)	622 (35.13%)
Bacteria/plaque: marked	306	7.23 (7.56)	4.83 (1.05–9.99)	107 (35.95%)	84 (27.51%)	115 (37.55%)
not marked	1498	7.19 (8.05)	3.96 (1.16–9.49)	535 (35.68%)	447 (29.81%)	517 (34.51%)
Hereditary: marked	33	9.48 (10.23)	5.37 (0.66–13.70)	12 (34.91%)	8 (23.90%)	14 (41.19%)
not marked	1772	7.16 (7.92)	4.00 (1.15–9.50)	630 (35.57%)	523 (29.52%)	618 (34.91%)
Other: Gingivitis/inflammation: marked	112	7.11 (8.87)	3.69 (0.63–7.64)	42 (37.70%)	37 (33.36%)	32 (28.94%)
not marked	1693	7.21 (7.91)	4.11 (1.17–9.67)	599 (35.42%)	494 (29.16%)	600 (35.42%)
Other reason: marked	241	6.64 (7.23)	4.10 (1.27–9.08)	89 (36.77%)	73 (30.08%)	80 (33.15%)
not marked	1564	7.29 (8.08)	4.04 (1.12–9.64)	553 (35.37%)	458 (29.32%)	552 (35.31%)
3. Oral hygiene habits						
Mouthwash use: Yes	1184	7.20 (8.00)	4.13 (1.21–9.39)	415 (35.02%)	365 (30.81%)	405 (34.17%)
No	621	7.19 (7.92)	3.95 (1.01–9.83)	227 (36.59%)	166 (26.77%)	227 (36.64%)
Toothbrushing: ≥twice/day	1606	7.07 (7.91)	3.97 (1.14–9.33)	577 (35.91%)	485 (30.23%)	544 (33.86%)
<twice/day	199	8.23 (8.36)	5.34 (1.22–11.73)	65 (32.72%)	46 (22.89%)	88 (44.39%)
Interdental brush use: Yes	25	5.54 (4.70)	3.84 (0.68–7.99)	9 (35.37%)	8 (32.12%)	8 (32.51%)
No	1780	7.22 (8.00)	4.06 (1.14–9.60)	633 (35.56%)	523 (29.38%)	624 (35.06%)
Flossing: Yes	730	6.32 (7.02)	3.64 (1.01–8.24)	280 (38.35%)	225 (30.85%)	225 (30.79%)
No	1074	7.80 (8.51)	5.00 (1.25–10.70)	362 (33.66%)	306 (28.45%)	407 (37.89%)
Toothpick use: Yes	379	8.10 (8.42)	5.10 (1.31–11.54)	125 (33.12%)	99 (26.22%)	154 (40.66%)
No	1426	6.96 (7.83)	3.93 (1.10–9.12)	516 (36.21%)	432 (30.27%)	478 (33.52%)
4. Self–reported gum bleeding						
Yes	1040	8.07 (8.46)	5.18 (1.54–11.05)	323 (31.09%)	309 (29.72%)	408 (39.19%)
No	765	6.02 (7.10)	3.42 (0.00–7.86)	318 (41.64%)	222 (29.01%)	224 (29.34%)

After adjusting for potential confounders (Table 3), participants who indicated no, little, or adequate knowledge about gum health had higher odds of being in the top OHIP tertile, compared to those who self-reported their knowledge as “good”. Compared to the reference (highest knowledge level) group, the odds appeared to be increasingly higher with decreasing level of knowledge; participants with no knowledge had more than double the odds of being in the top tertile (multivariable-adjusted OR=2.38, 95% CI: 1.59, 3.54).

**Table 3:** Multivariate odds ratios (ORs) and 95% confidence intervals (CIs) for tertiles of quality of life score (lowest tertile was used as the reference), according to predictors*, among all participants

Predictors	Tertile 2 vs Tertile 1 (ref.)		Tertile 3 vs Tertile 1 (ref.)	
	OR (95% CI)	p-value	OR (95% CI)	p-value
1. Self-assessed knowledge about gum health (WtN=1805)				
None	1.26 (0.80; 1.25)	0.3221	2.38 (1.59; 3.54)	<0.0001***
Little	1.30 (0.78; 2.15)	0.3182	1.82 (1.19; 2.78)	0.0055***
Adequate	1.50 (1.00; 2.26)	0.0500	1.68 (1.11; 2.57)	0.0154***
Good (ref.)	1.0	–	1.0	–
2. In your opinion, gums bleed because…, mark all that apply** (WtN=1805)				
Don’t know	0.71 (0.49; 1.03)	0.0689	0.63 (0.34; 1.15)	0.1318
Bad toothbrushing/hygiene	1.19 (0.80; 1.76)	0.3973	1.02 (0.54; 1.94)	0.9414
Smoking	0.83 (0.34; 2.03)	0.6774	0.47 (0.23; 0.96)	0.0376***
Bacteria/plaque	0.81 (0.51; 1.28)	0.3590	0.82 (0.49; 1.35)	0.4313
Hereditary	0.79 (0.40; 1.58)	0.5022	1.19 (0.65; 2.17)	0.5717
Other: gingivitis/inflammation	0.91 (0.49; 1.71)	0.7693	0.63 (0.33; 1.21)	0.1652
Other reason	0.86 (0.48; 1.53)	0.6048	0.71 (0.38; 1.35)	0.2974
3. Oral hygiene habits (WtN=1805)				
Mouthwash use, yes vs no (ref.)	1.21 (0.99; 1.49)	0.0661	1.11 (0.87; 1.40)	0.4074
Toothbrushing,≥twice /day vs <twice/day (ref.)	1.20 (0.77; 1.86)	0.4240	0.67 (0.48; 0.92)	0.0142***
Interdental brush use, yes vs no (ref.)	1.15 (0.51; 2.60)	0.7301	1.15 (0.47; 2.81)	0.7672
Flossing, yes vs no (ref.)	0.95 (0.74; 1.22)	0.6932	0.91 (0.67; 1.25)	0.5704
Toothpick use, yes vs no (ref.)	0.91 (0.66; 1.26)	0.5704	1.21 (0.86; 1.71)	0.2734
4. Self-reported gum bleeding (WtN=1805)				
Yes vs no (ref.)	1.43 (1.08; 1.90)	0.0136***	2.03 (1.60; 2.58)	<0.0001***
5. What do you do when your gums bleed, among those who self-reported gum bleeding (WtN=1040)				
Do nothing, yes vs no (ref.)	2.85 (0.55; 14.85)	0.2139	1.38 (0.39; 4.88)	0.6206
Brush more often, yes vs no (ref.)	3.63 (0.68; 19.27)	0.1308	2.85 (0.86; 9.44)	0.0870
Avoid touching the area, yes vs no (ref.)	3.59 (0.62; 20.84)	0.1539	2.26 (0.69; 7.39)	0.1781
Consult, yes vs no (ref.)	1.56 (0.18; 13.42)	0.6878	0.83 (0.11; 6.19)	0.8566

*Odds ratio estimates were obtained from a multinomial logistic regression model, with the listed variables as predictors and the three-level categorical OHIP score (tertile) as the outcome, using the lowest tertile (best summary quality of life score) as the reference. Models were adjusted for participants’ age (years), biological sex (male, female), smoking (3 categories: never, past, current), education (university, technical, none/basic), frequency of dental visits (only when there is a problem, at least once a year, never, missing), number of missing teeth, and location (Kingston, San Juan, Santo Domingo). **For this multiple-choice question, participants had the opportunity to choose more than one answer. In statistical analysis, participants who didn’t mark the answer (as correct) were used as the referent group. ***p-values were significant at the 0.05 level.

Participants reporting toothbrushing at least twice a day had 0.67 times the odds of being in the worst tertile for OHIP (vs best/lowest tertile), compared to those brushing less than twice a day (adjusted OR=0.67; 95% CI: 0.48; 0.92). Participants self-reporting gum bleeding had higher odds of being in the second tertile for quality of life (adjusted OR=1.43, 95% CI: 1.08, 1.90); they also had double the odds (adjusted OR=2.03, 95%CI: 1.60, 2.58) of being in the top tertile for OHIP (worst quality of life), compared to those who did not report bleeding.

When the original OHIP score was considered as the outcome for multivariate analysis, the results (Table 4) were similar to those from tertile analysis. Participants self-reporting no/little/adequate knowledge about gum health had statistically significantly higher OHIP scores, compared to those who reported “good” knowledge on the subject. Similarly, those self-reporting gum bleeding had statistically significantly higher OHIP scores after adjusting for potential confounders in multivariate regression analysis.

**Table 4:** Multivariate negative binomial regression coefficients (b), their standard errors, exponentiated coefficients with 95% confidence intervals (CI) for the summary quality of life score, according to categories of potential predictors*, among all participants

Model/Predictors	B	SE	exp(b) (95% CI)	p-value
1. Self-assessed knowledge about gum health (WtN=1805)				
None	0.2973	0.0973	1.35 (1.11; 1.63)	0.0022***
Little	0.2074	0.0882	1.23 (1.04; 1.46)	0.0187***
Adequate	0.1831	0.0924	1.20 (1.00; 1.44)	0.0475***
A lot (ref.)	–	–	–	–
2. In your opinion, gums bleed because…, mark all that apply** (WtN=1805)				
Don’t know	-0.1581	0.1162	0.85 (0.68; 1.07)	0.1736
Bad toothbrushing/hygiene	-0.0017	0.1115	1.00 (0.80; 1.24)	0.9880
Smoking	-0.2442	0.1956	0.78 (0.53; 1.15)	0.2118
Bacteria/plaque	-0.0704	0.1117	0.93 (0.75; 1.16)	0.5285
Hereditary	0.0426	0.1834	1.04 (0.73; 1.49)	0.8163
Other: gingivitis/inflammation	-0.1482	0.1344	0.86 (0.66; 1.12)	0.2701
Other reason	-0.1148	0.1260	0.89 (0.70; 1.14)	0.3622
3. Oral hygiene habits (WtN=1805)				
Mouthwash use, yes vs no (ref.)	0.0364	0.0519	1.03 (0.94; 1.15)	0.4838
Toothbrushing, ≥twice /day vs<twice/day (ref.)	-0.1393	0.0737	0.87 (0.75; 1.01)	0.0588
Interdental brush use, yes vs no (ref.)	0.0339	0.2076	1.03 (0.69; 1.55)	0.8703
Flossing, yes vs no (ref.)	-0.0291	0.0573	0.97 (0.87; 1.09)	0.6115
Toothpick use, yes vs no (ref.)	0.0577	0.0584	1.06 (0.94; 1.19)	0.3228
4. Self-reported gum bleeding (WtN=1805)				
Yes vs no (ref.)	0.2382	0.0495	1.27 (1.15; 1.40)	<0.0001***
**5. What do you do when your gums bleed, among those who self-reported gum bleeding (WtN=1040)**				
Do nothing, yes vs no (ref.)	0.1025	0.2668	1.11 (0.66; 1.87)	0.7007
Brush more often, yes vs no (ref.)	0.3211	0.2670	1.38 (0.82; 2.33)	0.2291
Avoid touching the area, yes vs no (ref.)	0.2376	0.2821	1.27 (0.73; 2.21)	0.3997
Consult, yes vs no (ref.)	-0.1096	0.3535	0.90 (0.45; 1.79)	0.7564

*Estimates were obtained from a negative binomial regression models, adjusted for participants’ age (years), biological sex (male, female), smoking (3 categories: never, past, current), education (university, technical, none/basic), frequency of dental visits (only when there is a problem, at least once a year, never, missing), number of missing teeth, and location (Kingston, San Juan, Santo Domingo). **For this multiple-choice question, participants had the opportunity to choose more than one answer. In statistical analysis, participants who didn’t mark the answer (as correct) were used as the referent group. ***p-values were significant at 0.05 level.

## DISCUSSION

The World Health Organization has stated that oral health is a key indicator of overall health, well-being, and quality of life.^24^ Our study used the Oral Health Impact Profile (OHIP-14) to assess the Oral Health Related Quality of Life among Caribbean Adults. The short form of OHIP with fourteen questions is widely used to evaluate the self-perception of oral health related quality of life (OHRQoL).

The OHIP-14 used in this study included adult individuals from three capital cities (Kingston, Santo Domingo and San Juan) of the participating Caribbean countries (Jamaica, Dominican Republic and Puerto Rico). The average oral health-related quality of life score was 7.2, and 41% of study participants indicated no knowledge of their gum health and were in poorest quality of life. This finding was similar to a study conducted in the United States where the researchers identified 34% of their participants received poor scores in oral health knowledge.^14^ The above mentioned study showed that the variation of OHIP-14 score among various geographical locations may be attributed to oral hygiene practices, different ethnicities, dental health seeking behaviour or awareness of oral health knowledge and practices. This study also found that participants who reported flossing and toothbrushing twice per day were more likely to be in lowest tertile of OHIP indicating better quality of life. This may be attributed to the fact that brushing is considered to be the most common and basic mode for getting optimal periodontal health. However, the results may vary with intervals, duration and type of brushing. Our multivariable-adjusted regression analyses showed that participants who brush at least twice a day had 0.67 times the odds of being in the worst tertile for OHIP than those brushing less than twice a day. Brushing and interdental cleaning practices promotes removal of plaque from tooth surface and are essential to prevent the development of gum and periodontal diseases. However, incorrect brushing habits may traumatize gingival tissue which may lead to gingival bleeding and gingival recession. In a recent study it was reported that interdental cleaning with dental floss can be effective but difficult to use and technique-sensitive for most of the patients. Additionally, they reported that approximately 30%-60% of health information is forgotten within one hour, and 50% of health recommendations were not followed. Therefore, the authors recommended incorporating psychosocial aspects of behavioural change in well-established counseling strategies, such as motivational interviewing to improve patient outcomes.24 Thus, professional oral health care advice and prevention strategies such as routine oral self-care and timely visits to the dentist should be provided in a continuous manner to promote correct oral health practices and prevent hard toothbrushing related oral health problems and consequences.

In our study, among the participants who reported toothpick using habits, 40.66% were in the top tertile of OHIP with poor quality of life. In addition, 39.19% of participants who reported gum bleeding, were also included in the top tertile of OHIP with poor quality of life. On the contrary, other authors found a lack of statistical evidence for a relationship between periodontal disease status and OHRQoL.^10^^,^^23^

Various studies reported that mouth rinses, floss and interdental brushes are frequently used in auxiliary dental hygiene practices.^2^^,^^20^^,^^21^ However, the use of toothpicks in the current study may be related to low socioeconomic status of the participants. Higher gum bleeding may be attributed to improper brushing methods and/or incorrect use of toothpick due to recurrent tiny micro-abrasions in the gingival tissues. A study that investigated gingival bleeding and toothbrushing showed a significant reduction of bleeding on probing sites after use of toothbrushing and interdental cleaning.^2^

One of the major findings of this study was the strong association between the self-assessed oral health knowledge and OHIP. The odds of being in the top OHIP tertile were significantly higher among participants indicating no, little or adequate knowledge about gum health, compared to those who self-reported as knowing “a lot” about gum health, after adjusting for potential confounders. These findings indicate that association of communicative oral health literacy versus existing knowledge on oral health practice is crucial in improving oral health outcomes. Thus, Oral Health Literacy (OHL) is highly important for oral health outcomes, since low OHL has been associated with lack of use of dental services, failure to adhere appropiate medical instructions, and poor self-oral health care management skills, all of which influence the quality of life and oral health problems.^3^ Various studies indicate that disease prevention and health promotion programs in developing and developed countries are needed, in order to improve oral health conditions, and particularly periodontal status. These programs would contribute to prevent the appearance of more complex and severe oral diseases, maintaining a good quality of life and general health of the individuals.^4^^,^^11^

This study had several strengths, as well as limitations. The cross-sectional design of this study did not allow for establishment of temporality between the variables. Moreover, the health-related behaviour data were self-reported and are therefore not verifiable. On the other hand, this large population-based study included a representative and balanced sample of participants from three different countries in the Caribbean. A validated questionnaire was used for assessment of oral health-related quality of life, with the resulting evidence contributing to the growing body of literature on this important public health topic. Future investigations are needed to develop strategies that improve Caribbean adults’ oral health knowledge, practices, and status.

## CONCLUSION

The findings from this survey suggest that gaps in oral health knowledge need to be addressed for improving Oral Health Related Quality of Life in Caribbean adults. Improper brushing methods, interdental hygiene practices are directly related to poor oral health and subsequently leads to poor quality of life. Thus, promotion of a positive attitude towards oral health practices is the key for bringing better oral health among Caribbean adults. Hence, oral health associations, health care providers, the private sector, policy makers, and ministries of health should collectively work on developing sound strategies for improving oral health practices not just increasing the access for dental/oral health centers but also strictly focus on raising awareness on self-oral care practices and dental visits.

## Figures and Tables

**Table:** Oral Health Impact Profile (OHIP) QUESTIONNAIRE

ORAL HEALTH IMPACT PROFILE						
	Name				Date	
	HOW OFTEN have you had the problem during the last year? (Circle your answer)					
1. Have you had trouble pronouncing any words because of problems with your teeth, mouth or dentures?	**VERY OFTEN**	**FAIRLY OFTEN**	**OCCASIONALLY**	**HARDLY EVER**	**NEVER**	**DON’T KNOW**
2. Have you felt that your sense of taste has worsened because of problems with your teeth, mouth or dentures?	**VERY OFTEN**	**FAIRLY OFTEN**	**OCCASIONALLY**	**HARDLY EVER**	**NEVER**	**DON’T KNOW**
3. Have you had painful aching in your mouth?	**VERY OFTEN**	**FAIRLY OFTEN**	**OCCASIONALLY**	**HARDLY EVER**	**NEVER**	**DON’T KNOW**
4. Have you found it uncomfortable to eat any foods because of problems with your teeth, mouth or dentures?	**VERY OFTEN**	**FAIRLY OFTEN**	**OCCASIONALLY**	**HARDLY EVER**	**NEVER**	**DON’T KNOW**
5. Have you been self-conscious because of your teeth, mouth or dentures?	**VERY OFTEN**	**FAIRLY OFTEN**	**OCCASIONALLY**	**HARDLY EVER**	**NEVER**	**DON’T KNOW**
6. Have you felt tense because of problems with your teeth, mouth or dentures?	**VERY OFTEN**	**FAIRLY OFTEN**	**OCCASIONALLY**	**HARDLY EVER**	**NEVER**	**DON’T KNOW**
7. Has your diet been unsatisfactory because of problems with your teeth, mouth or dentures?	**VERY OFTEN**	**FAIRLY OFTEN**	**OCCASIONALLY**	**HARDLY EVER**	**NEVER**	**DON’T KNOW**
8. Have you had to interrupt meals because of problems with your teeth, mouth or dentures?	**VERY OFTEN**	**FAIRLY OFTEN**	**OCCASIONALLY**	**HARDLY EVER**	**NEVER**	**DON’T KNOW**
9. Have you found it difficult to relax because of problems with your teeth, mouth or dentures?	**VERY OFTEN**	**FAIRLY OFTEN**	**OCCASIONALLY**	**HARDLY EVER**	**NEVER**	**DON’T KNOW**
10. Have you been a bit embarrassed because of problems with your teeth, mouth or dentures?	**VERY OFTEN**	**FAIRLY OFTEN**	**OCCASIONALLY**	**HARDLY EVER**	**NEVER**	**DON’T KNOW**
11. Have you been a bit irritable with other people because of problems with your teeth, mouth or dentures?	**VERY OFTEN**	**FAIRLY OFTEN**	**OCCASIONALLY**	**HARDLY EVER**	**NEVER**	**DON’T KNOW**
12. Have you had difficulty doing your usual jobs because of problems with your teeth, mouth or dentures?	**VERY OFTEN**	**FAIRLY OFTEN**	**OCCASIONALLY**	**HARDLY EVER**	**NEVER**	**DON’T KNOW**
13. Have you felt that life in general was less satisfying because of problems with your teeth, mouth or dentures?	**VERY OFTEN**	**FAIRLY OFTEN**	**OCCASIONALLY**	**HARDLY EVER**	**NEVER**	**DON’T KNOW**
14. Have you been totally unable to function because of problems with your teeth, mouth or dentures?	**VERY OFTEN**	**FAIRLY OFTEN**	**OCCASIONALLY**	**HARDLY EVER**	**NEVER**	**DON’T KNOW**

**Supplement II:**
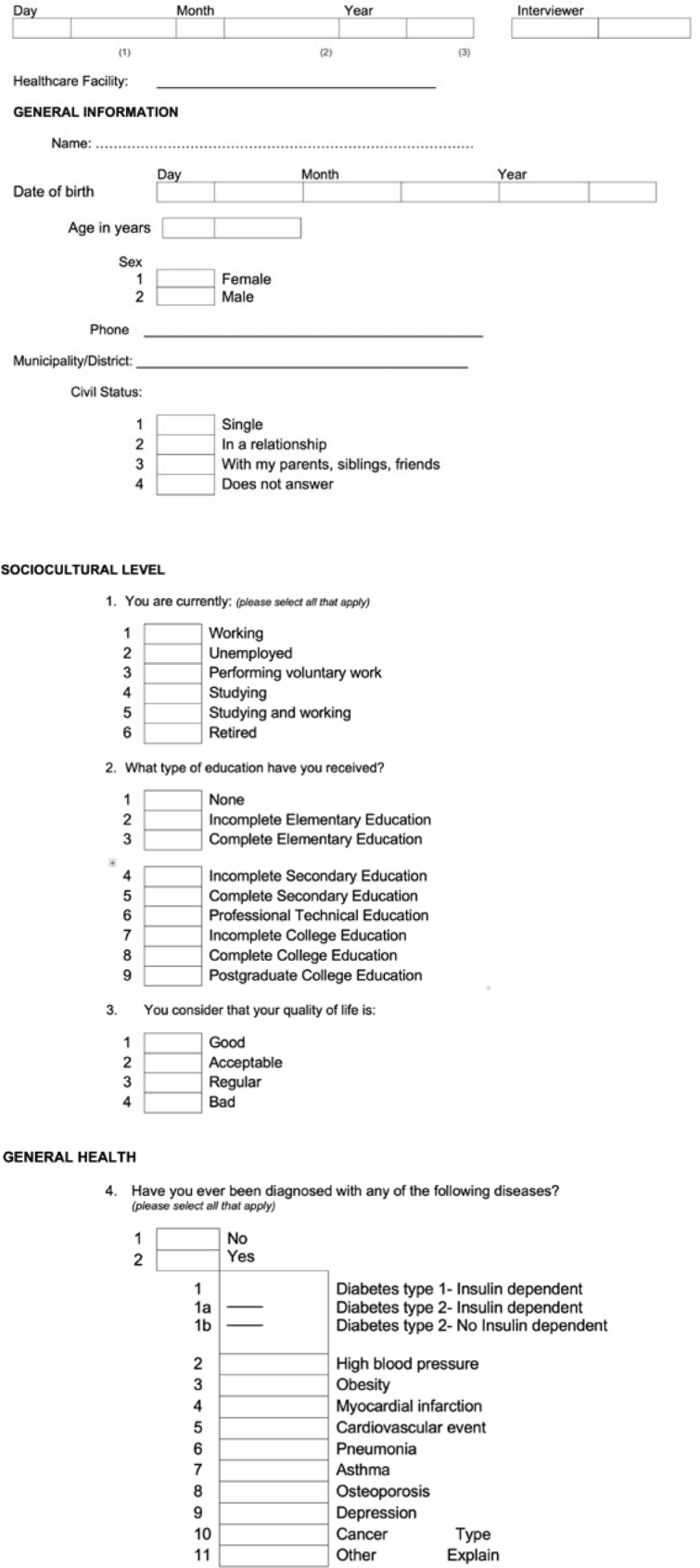

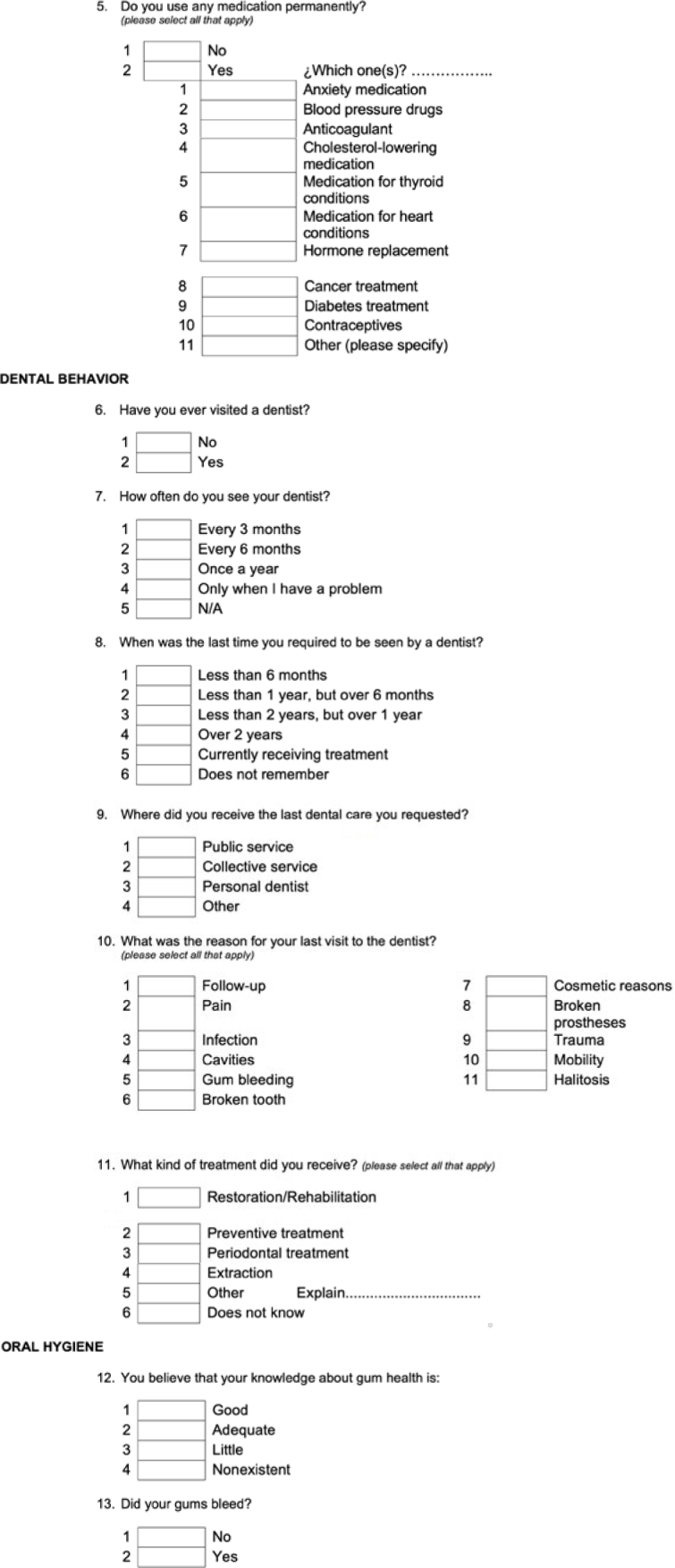

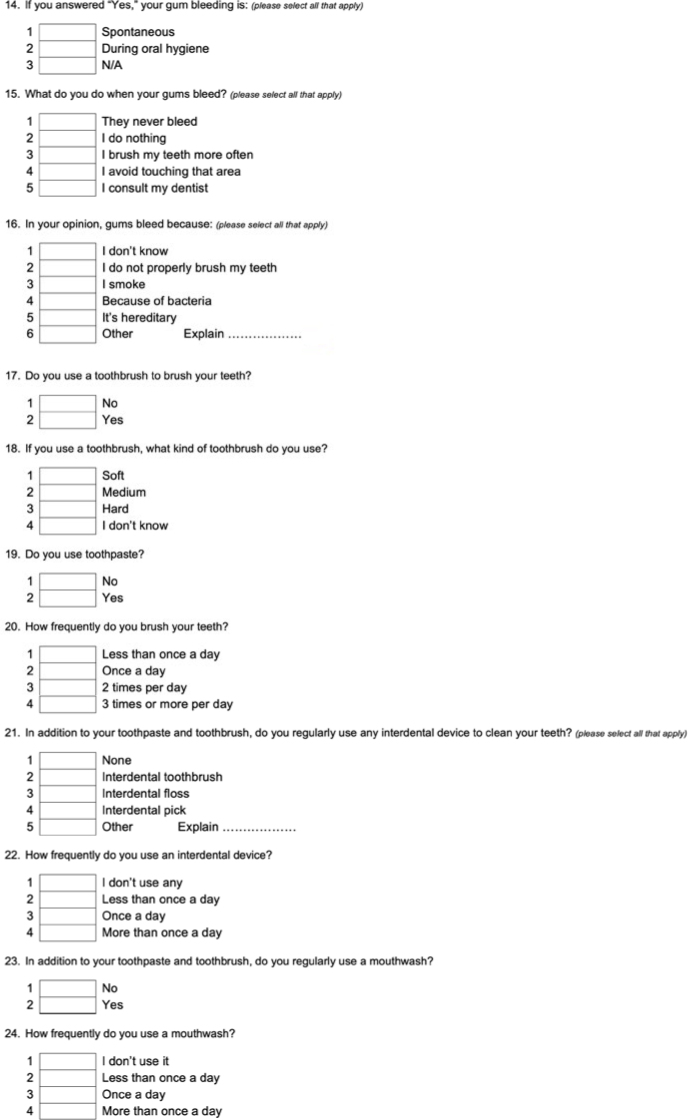

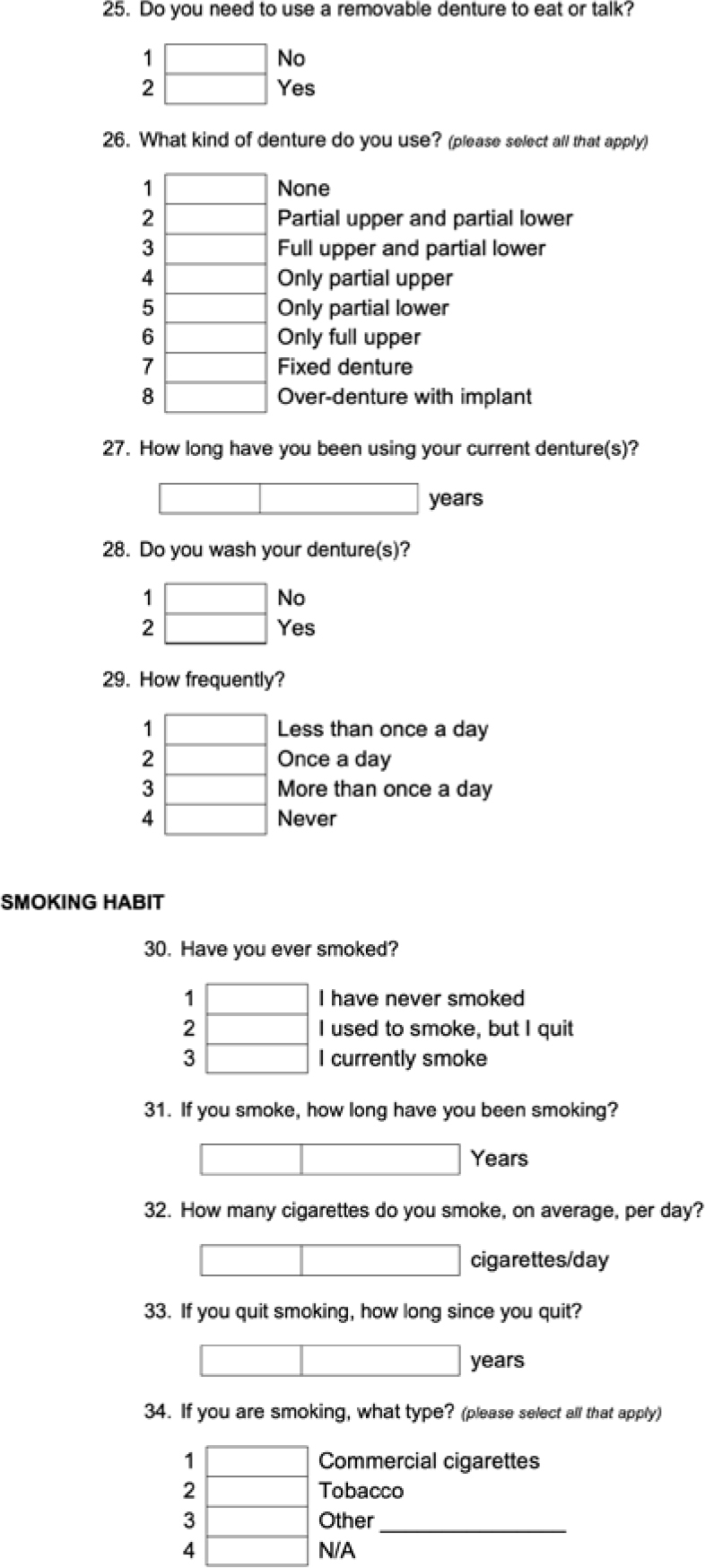
MEDICAL HISTORY / ORAL HEALTH QUESTIONNAIRE
